# Unique protein expression signatures of survival time in kidney renal clear cell carcinoma through a pan-cancer screening

**DOI:** 10.1186/s12864-017-4026-6

**Published:** 2017-10-03

**Authors:** Guangchun Han, Wei Zhao, Xiaofeng Song, Patrick Kwok-Shing Ng, Jose A. Karam, Eric Jonasch, Gordon B. Mills, Zhongming Zhao, Zhiyong Ding, Peilin Jia

**Affiliations:** 1 Center for Precision Health, School of Biomedical Informatics, The University of Texas Health Science Center at Houston, 7000 Fannin St., Suite 820, Houston, TX 77030 USA; 2 Department of Systems Biology, University of Texas MD Anderson Cancer Center, Houston, TX 77030 USA; 3 Department of Biomedical Engineering, Nanjing University of Aeronautics and Astronautics, Nanjing, Jiangsu 211106 China; 4 Institute for Personalized Cancer Therapy, The University of Texas MD Anderson Cancer Center, Houston, TX 77030 USA; 5 Department of Urology, Division of Surgery, The University of Texas MD Anderson Cancer Center, Houston, TX 77030 USA; 6 Department of Genitourinary Medical Oncology, The University of Texas MD Anderson Cancer Center, Houston, TX 77030 USA; 7 Human Genetics Center, School of Public Health, The University of Texas Health Science Center at Houston, Houston, TX 77030 USA

**Keywords:** Pan-cancer screening, Kidney renal clear cell carcinoma (KIRC), Reverse phase protein Array (RPPA), Protein expression, Prognostic biomarker

## Abstract

**Background:**

In 2016, it is estimated that there will be 62,700 new cases of kidney cancer in the United States, and 14,240 patients will die from the disease. Because the incidence of kidney renal clear cell carcinoma (KIRC), the most common type of kidney cancer, is expected to continue to increase in the US, there is an urgent need to find effective diagnostic biomarkers for KIRC that could help earlier detection of and customized treatment strategies for the disease. Accordingly, in this study we systematically investigated KIRC’s prognostic biomarkers for survival using the reverse phase protein array (RPPA) data and the high throughput sequencing data from The Cancer Genome Atlas (TCGA).

**Results:**

With comprehensive data available in TCGA, we systematically screened protein expression based survival biomarkers in 10 major cancer types, among which KIRC presented many protein prognostic biomarkers of survival time. This is in agreement with a previous report that expression level changes (mRNAs, microRNA and protein) may have a better performance for prognosis of KIRC. In this study, we also identified 52 prognostic genes for KIRC, many of which are involved in cell-cycle and cancer signaling, as well as 15 tumor-stage-specific prognostic biomarkers. Notably, we found fewer prognostic biomarkers for early-stage than for late-stage KIRC. Four biomarkers (the RPPA protein IDs: FASN, ACC1, Cyclin_B1 and Rad51) were found to be prognostic for survival based on both protein and mRNA expression data.

**Conclusions:**

Through pan-cancer screening, we found that many protein biomarkers were prognostic for patients’ survival in KIRC. Stage-specific survival biomarkers in KIRC were also identified. Our study indicated that these protein biomarkers might have potential clinical value in terms of predicting survival in KIRC patients and developing individualized treatment strategies. Importantly, we found many biomarkers in KIRC at both the mRNA expression level and the protein expression level. These biomarkers shared a significant overlap, indicating that they were technically replicable.

**Electronic supplementary material:**

The online version of this article (doi:10.1186/s12864-017-4026-6) contains supplementary material, which is available to authorized users.

## Background

Cancer remains a leading cause of death in the United States [1]. The identification of effective prognostic biomarkers for patient outcomes can greatly improve early cancer detection and treatment strategy selection. Accordingly, locating survival biomarkers has been a major goal during recent decades. Thanks to revolutionary advances in high-throughput biotechnology, especially microarrays and next-generation sequencing, the volume of biomedical data available to researchers has increased exponentially. Many large cancer research programs have been launched or already completed, aiming at deciphering the complex molecular pattern in cancer. These programs include The Cancer Genome Atlas (TCGA), The Cancer Proteome Atlas (TCPA) [2], and The International Cancer Genome Consortium (ICGC). Launched in 2005, TCGA was dedicated to characterize samples of more than 30 types of cancer, generating enormous amounts of comprehensive genetic, epigenetic, transcriptomic, and proteomic data that enable researchers to systematically investigate biomarkers across all the major types of cancer.

There are many types of potential biomarkers for cancer. DNA alterations are a major type of biomarker candidates, including single nucleotide variants (SNVs), small insertions and deletions (indels), copy number variations (CNVs), and structural variants (SVs). Most successful examples of DNA mutations as biomarkers were found in well-studied cancer genes, such as *EGFR* [3], *KRAS* [4], and *BRAF* [5]. Gene expression patterns were commonly used to predict patient outcomes when microarrays were widely used to measure mRNA expression [6], and this trend has continued now that sequencing, including genome sequencing and transcriptome sequencing, has become the methodology of choice. Many studies have been conducted to discover mRNA biomarkers in numerous cancer types [7–11], yet few results have been replicated in independent cohorts. Protein expression has also been examined for its biomarker potential, although proteomic technology is well behind that of DNA or RNA assays at the genomic scale. Reverse-phase protein arrays (RPPAs) provide an efficient way to simultaneously quantify the expression level of many proteins [12], although they have not yet been used to survey all human proteins.

In 2016, it is estimated that there will be 62,700 new cases of kidney cancer in the United States — an increase of approximately 8% compared to 2010 — and that 14,240 patients will die from the disease [1, 13]. Because the incidence of kidney renal clear cell carcinoma (KIRC), the most common type of kidney cancer, is expected to continue increasing in the US [14], there is an urgent need to find effective prognostic biomarkers for KIRC. TCGA [15], as well as several other large-scale cancer genome studies [16–18], have systematically investigated KIRC’s molecular profiles in hundreds of samples, but to date, studies of somatic mutations in KIRC have found only a few potentially useful prognostic markers. These include the tumor-suppressor genes *VHL*, which is involved in the degradation of hypoxia inducible factor and is associated with both the sporadic and familial forms of KIRC [15]; *PBRM1*, a gene involved in chromatin remodeling [19, 20]; *BAP1*; and *PTEN*. Notably, in KIRC it is the tumor-suppressor genes that are most prone to mutation, whereas in other cancer types mutation is most common in oncogenes.

In this study, we conducted a systematic screening for somatic-mutation, mRNA-expression, and protein-expression prognostic biomarkers. We started with a pan-cancer screening, which helped us to discover only moderate numbers of significant biomarkers in most cancer types. Remarkably, our pan-cancer results highlighted an unusually large number of protein biomarkers in KIRC compared to other types of cancer. Therefore, we focused on the further analyses of these protein biomarkers in KIRC. Specifically, we explored their correlation with changes in mRNA expression, their co-expression patterns, their crosstalk with other domains of data, and their unique power to predict patient outcome in KIRC samples from specific stages of cancer. In summary, the present study provides a comprehensive overview of the protein biomarkers in KIRC.

## Results

### Analysis workflow

**Fig. 1:**
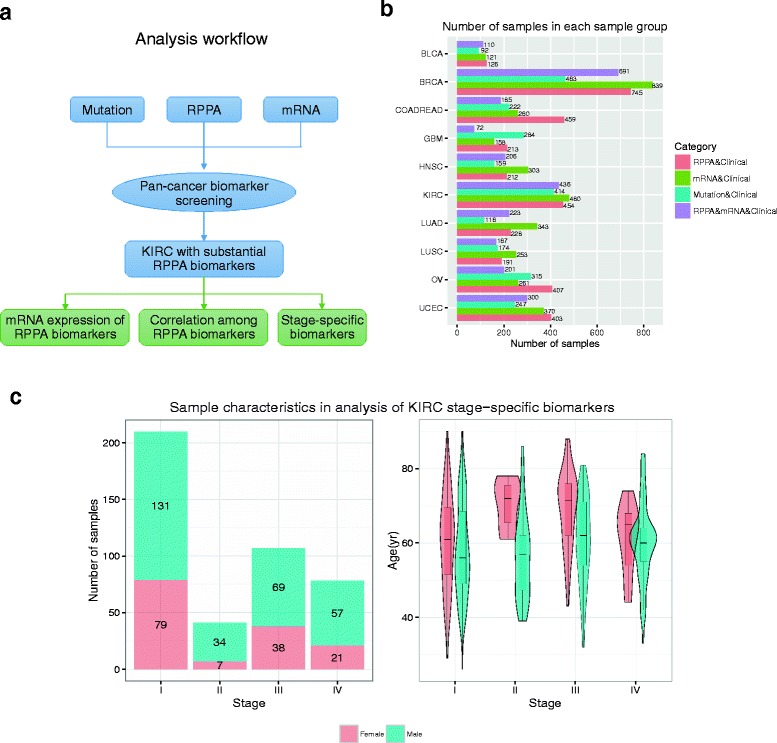
Analysis workflow and sample statistics. **a** Overview of analysis workflow. **b** Distribution of the number of samples for each of ten cancers screened for biomarkers prognostic for survival. **c** Characteristics of samples used in the process of identifying stage-specific KIRC biomarkers. Abbreviations for cancer names were provided in the main text

As is depicted in Fig. 1a, in this study, we first performed pan-cancer screening for survival biomarkers in 11 cancer types: bladder carcinoma (BLCA), breast invasive carcinoma (BRCA), colon adenocarcinoma (COAD), glioblastoma multiforme (GBM), head-neck squamous cell carcinoma (HNSC), kidney renal clear cell carcinoma (KIRC), lung adenocarcinoma (LUAD), lung squamous cell carcinoma (LUSC), ovarian cancer (OV), rectum adenocarcinoma (READ), and uterine corpus endometrial carcinoma (UCEC). We decided to combine the COAD and READ samples, collectively referred as COADREAD, in our analyses as the two cancer types are closely related [21]. Thereafter, we referred to our samples as being from ten cancer types.

In seeking prognostic biomarkers, we considered somatic mutations, mRNA expression, and protein expression. For each cancer type, we collected samples that had clinical information and corresponding genetic mutation data, gene expression data and protein expression data from TCGA or the cBio Cancer Genomics Portal [22]. Figure 1b shows the data available for the different types of cancer. Unless otherwise stated, for each type of survival analysis, we utilized sample subsets with two-dimensional data. For example, samples with somatic mutation and clinical data were used for somatic mutation biomarker analysis, samples with mRNA expression and clinical data (the MC group) were used for mRNA biomarker analysis; and samples with protein expression and clinical data (the PC group) were used for protein biomarker analysis. To enable comparisons between mRNA and protein expression, we also defined a subset of samples (the PMC group) that had protein expression data, mRNA expression data, and clinical data. The number of samples for each cancer type used in our analysis ranged from 72 to 839. Survival analysis was conducted for each gene, and the chi-square test (for category parameters, such as mutation data) or Cox proportional hazards regression model (for continuous parameters, such as mRNA and protein expression data) was employed to identify significant biomarkers. For all three types of biomarkers, we characterized a false discovery rate (FDR) < 0.05 as significant, where FDR was estimated using Benjamini and Hochberg’s method [23] of making multiple corrections of the *p* values in statistical tests (e.g., the chi-square test).

### Screening for survival biomarkers using somatic mutation data

For somatic mutations, we only considered non-silent SNVs and indels (i.e., changing amino acids) and mapped these variants to genes using ANNOVAR [24]. We screened mutation biomarkers for ten cancer types. The results for each cancer are summarized in Table 1. At FDR < 0.05, the number of significant mutation biomarkers ranged from 0 and 31. Three cancer types (BLCA, COADREAD, and LUAD) had no prognostic biomarkers from the somatic mutations. Six cancer types had <10 mutation-based survival biomarkers: BRCA, GBM, HNSC, LUSC, OV and KIRC. We identified six prognostic biomarkers in BRCA (*ZNF536*, *p*_adjust_ = 9.6 × 10^−4^; *BRWD1*, *p*_adjust_ = 0.02; *CENPE*, *p*_adjust_ = 0.03; *MED12*, *p*_adjust_ = 0.03; *DLGAP4*, *p*_adjust_ = 0.03 and *FAM179A*, *p*_adjust_ = 0.04 where *p*_adjust_ indicates multiple-testing-corrected *p* values calculated using the Benjamini and Hochberg method). For GBM, we also detected six prognostic biomarkers, including *ZFHX3* (*p*_adjust_ = 8.2 × 10^−5^), *CTTNBP2* (*p*_adjust_ = 2.2 × 10^−4^), *ZNF99* (*p*_adjust_ = 0.02), *CARD11* (*p*_adjust_ = 0.03), *IGFN1* (*p*_adjust_ = 0.03) and *WDR63* (*p*_adjust_ = 0.04). We identified four prognostic biomarkers in HNSC (*DNAH17*, *p*_adjust_ = 3.9 × 10^−3^; *RSPH4A*, *p*_adjust_ = 6.7 × 10^−3^; *CILP*, *p*_adjust_ = 0.02 and *NBEA*, *p*_adjust_ = 0.02). LUSC had six significant biomarkers, including *UGT8* (*p*_adjust_ = 1.5 × 10^−9^), *DDC* (*p*_adjust_ = 3.9 × 10^−4^), *SOGA2* (*p*_adjust_ = 1.6 × 10^−3^), *SEPT14* (*p*_adjust_ = 1.6 × 10^−3^), *HERC6* (*p*_adjust_ = 0.01) and *ZNF81* (*p*_adjust_ = 0.02). OV had one survival biomarkers: *PLB1* (*p*_adjust_ = 0.04). In KIRC, we found only one mutation biomarker, *MTHFD1* (*p*_adjust_ = 0.01), which was mutated in five of 414 samples. UCEC had >10 mutation biomarkers. Among all the cancers we examined, UCEC had the largest number of SNV survival biomarkers with somatic mutations in 31 genes. The strongest prognostic biomarkers in UCEC were *GPR124* (*p*_adjust_ = 8.1 × 10^−8^), *KCNJ4* (*p*_adjust_ = 4.0 × 10^−6^), *TFIP11* (*p*_adjust_ = 1.8 × 10^−5^), *YIF1A* (*p*_adjust_ = 1.5 × 10^−4^), *SLC22A6* (*p*_adjust_ = 8.9 × 10^−4^), and *FSD1L* (*p*_adjust_ = 1.1 × 10^−3^). Five of 247 UCEC samples carried *GPR124* mutations. All five patients from whom the tested samples were taken had survival times of less than 10 months, substantially shorter than the 34-month average survival time seen in patients whose samples did not contain mutations in *GPR124*.

**Table 1:** Results of pan-cancer screening for prognostic biomarkers

Cancer type	Mutation analysis			RPPA analysis			mRNA analysis		
	Samples (N)	Genes (N)	Genes (N) (chi-square test, FDR < 0.05)	Samples (N)	Genes (N)	Genes (N) (log-rank test, FDR < 0.05)	Samples (N)	Genes (N)	Genes (N) (log-rank test, FDR < 0.05)
BLCA	92	63	0	110	17	2	110	9	0
BRCA	463	67	6	691	31	10	691	20	0
COADREAD	222	62	0	185	2	0	185	6	0
GBM	284	31	6	72	32	1	72	14	1
HNSC	159	50	4	206	17	0	206	12	0
KIRC	414	32	1	436	101	85	436	89	84
LUAD	116	59	0	223	15	0	223	30	1
LUSC	174	173	6	167	15	0	167	12	0
OV	315	14	1	201	18	0	201	7	0
UCEC	247	472	31	300	26	3	300	39	5

### Pan-Cancer screening for survival biomarkers using protein expression data

To find other potential survival biomarkers, we next screened protein expression data measured by the RPPA platform. According to TCGA data, the RPPA assay investigated 187 RPPA antibodies targeting 155 proteins. The original study of cancer functional proteomics had categorized these proteins into ten pathways [2]. The number of samples for each cancer type ranged from 126 (BLCA) to 745 (BRCA) in the PC sample group and from 72 (GBM) to 691 (BRCA) in the PMC sample group. We performed a Cox proportional hazards regression on the protein expression data from both sample groups (of note, the PMC sample group is a subset of the PC sample group). We performed the analysis on the PMC sample group for all ten cancer types. Surprisingly, we found that KIRC had the largest number of prognostic protein biomarkers (85 of 187, 45%, Table 1). Among the other nine cancer types, BRCA had the second most protein biomarkers (ten).

**Fig. 2:**
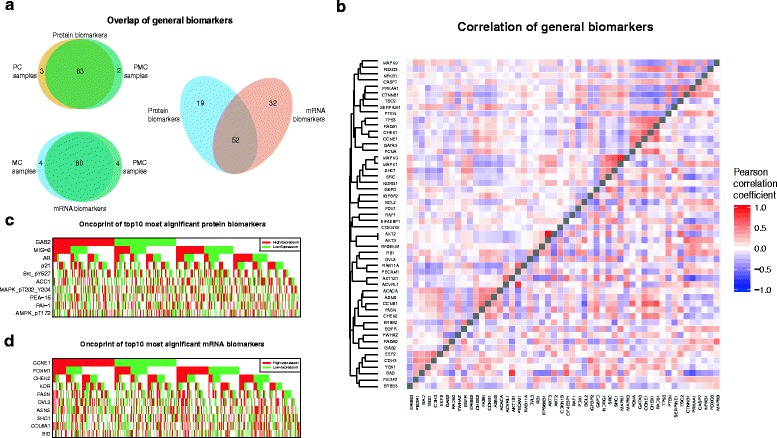
Protein prognostic biomarkers in KIRC. **a** The number of prognostic biomarkers identified in each sample group and the count of their overlapped markers (see details in main text). **b** Heatmap of Pearson’s correlation coefficient. The upper triangle of the heatmap shows correlation of protein expression, while the lower triangle shows correlation of mRNA expression. Biomarkers were clustered based on correlation of protein expression. **c** Oncoprints of top ten prognostic protein biomarkers. **d** Oncoprints of top ten prognostic mRNA biomarkers

The results above changed slightly when we conducted the analyses on the PC group. When the PC sample group was compared to the PMC sample group, the number of significant biomarkers increased for KIRC (86 in 454 PC samples vs. 85 in 436 PMC samples), OV (14 in 407 PC samples vs. 0 in 201 PMC samples), and UCEC (7 in 403 PC samples vs. 3 in 300 PMC samples). In addition, we examined the impact of sample size on prediction of biomarkers across cancer types. For example, in the PC sample group, the sample sizes for both UCEC (*n* = 403) and OV (*n* = 407) were similar to the sample size for KIRC (*n* = 454), but there were only 14 prognostic protein biomarkers for UCEC and seven for OV, substantially less than the 86 for KIRC. This confirmed that the large number of prognostic biomarkers we found in KIRC was only slightly attributable to the size of the sample. Moreover, there was substantial overlap in the protein biomarkers found in different sample groups. As shown in Fig. 2a, we compared the 85 KIRC protein biomarkers obtained using the PMC sample group and the 86 biomarkers found in the PC sample group. A total of 83 biomarkers overlapped, further confirming that the sample size had a very small impact on the biomarkers we found in KIRC.

Overall, the screening results showed that KIRC is different from all other cancer types in terms of the number of protein biomarkers of patient survival. The protein biomarkers in KIRC are also different from other types of biomarkers, such as those obtained using mutation data. Since most driver mutations previously reported in KIRC are in tumor-suppressor genes (i.e., are of limited clinical use), our finding of a large number of protein biomarkers has important implications for the development of precision medicine approaches to treat patients with KIRC. Hereafter, we refer to the 85 RPPA biomarkers found in the PMC sample group as the KIRC protein biomarkers and utilized them in subsequent analyses.

### Prognostic capabilities of mRNA biomarkers

Having observed that KIRC has a surprisingly large number protein biomarkers prognostic for survival, we next explored the mRNA expression levels of these protein biomarkers to determine whether the protein biomarkers’ mRNA counterparts were also useful for prognosis. The consistency between mRNA biomarkers and protein biomarkers would presumably rely on the correlation between mRNA and protein expression of each gene, which was subjected to many factors, such as post-transcriptional regulation, mRNA and protein degeneration, the corresponding technology in data generation, the heterogeneity of the cancer samples, and the clinical treatment the patients from whom the samples were obtained had received. In this work, we investigated the mRNA expression of the 155 proteins measured in the RPPA platform. We found that 148 of these proteins had mRNA-expression data available in TCGA pan-cancer RNA-sequencing data. Thus, following an analysis strategy similar to that we had used in analyzing protein-expression data, we performed a Cox proportional hazards regression on the mRNA expression data for two different sample groups: a PMC group with protein-expression, mRNA-expression, and clinical data, and an MC group that had mRNA-expression and clinical data.

Screening for prognostic mRNA biomarkers using the PMC sample group (*n* = 436) yielded 84 significant mRNA biomarkers for KIRC (84/148 = 57%, Table 1). The most significant prognostic mRNA biomarkers included *CCNE1* (*p*_adjust_ = 1.4 × 10^−11^), *FOXM1* (*p*_adjust_ = 7.8 × 10^−11^), *CHEK2* (*p*_adjust_ = 9.8 × 10^−11^), *KDR* (*p*_adjust_ = 2.4 × 10^−10^), and *FASN* (*p*_adjust_ = 2.9 × 10^−10^). In strong contrast, we did not find significant prognostic mRNA biomarkers for BLCA, BRCA, COADREAD, HNSC, LUSC, or OV. There was one mRNA biomarker in GBM and one in LUAD, and there were five in UCEC. We then performed the analysis using the MC sample group. Likely due to an increase in sample size, we observed a few more significant mRNA biomarkers in some cancer types, e.g., 11 in HNSC, 18 in LUAD, and 15 in UCEC. However, the number of mRNA biomarkers in KIRC was still substantially higher than that in any other cancer type, consistent with our findings using the protein expression data. Hereafter, we refer to the 84 mRNA prognostic biomarkers found in the KIRC PMC sample group as the KIRC mRNA biomarkers and used them in subsequent analyses.

### Overlap between protein biomarkers and mRNA biomarkers in KIRC

We next examined the correlation between KIRC’s protein and mRNA biomarkers in KIRC. The 85 protein biomarkers, which were antibodies, targeted 79 unique proteins, 71 of which had mRNA measurements in TCGA pan-cancer RNA-seq data. A total of 52 genes overlapped between the protein and mRNA biomarkers, corresponding to 62 antibodies and 52 mRNA genes (Fig. 2a). The overlapping genes were significantly overrepresented with protein biomarkers (*p =* 8.7 × 10^−5^, hypergeometric test). Hereafter, these 52 overlapped biomarkers (Additional file 1) are referred to as the general prognostic biomarkers of KIRC.

To explore the functions of these 52 genes, we conducted a pathway enrichment analysis using both the canonical Kyoto Encyclopedia of Genes and Genomes (KEGG) annotations and the ten pathways originally targeted by the RPPA platform [2] (Additional file 2). Our results showed that the overlapping genes were involved in the KEGG’s cancer-related signaling transduction pathways (hsa05215: prostate cancer, *p =* 2.2 × 10^−3^; hsa05223: non-small cell lung cancer, *p =* 0.024; and hsa04012: ErbB signaling pathway, *p =* 0.029). In addition, a previous study curated ten pathways that are covered by the antibodies measured in the RPPA platform [2]. In examining these custom-defined pathways, we found that the cell-cycle pathway was particularly enriched in our overlapping biomarkers (19/52 = 36.5%, *p =* 0.03). In summary, we found many prognostic biomarkers for KIRC at both the mRNA- and protein-expression levels. These biomarkers overlapped significantly and were enriched in cancer-related pathways, which suggested their potential clinical value.

### The large number of KIRC biomarkers was not due to co-expression

We next asked whether the biomarkers we had observed represented distinct expression patterns or whether they were driven by a few unique processes, while others were only co-expression partners. We used the Pearson Correlation Coefficient (PCC) to explore the co-expression pattern and calculated it for the 52 overlapping genes using protein-expression and mRNA-expression data, respectively (Fig. 2b). As shown in the heatmaps of mRNA expression (the lower triangle in Fig. 2b) and protein expression (the upper triangle in Fig. 2b), the majority of gene pairs showed low co-expression at both the mRNA and the protein levels. For example, only 28 (2.11%) of the 1326 gene pairs had a PCC value >0.5 for protein expression, indicating a strong lack of correlation among the 52 genes. In addition, there was weak consistency observed between the mRNA and protein co-expression patterns, as shown in Fig. 2b. Had there been strong consistency, the heatmap would have been mirrored along the diagonal line.

We also compared the 52 overlapped genes with other genes used in the same platform for their consistency between mRNA and protein expression. This allowed us to examine whether these biomarker genes exhibited especially high correlations at the mRNA and protein levels. We compared the protein-mRNA expression correlations of the 52 biomarkers with the correlations of other genes whose encoding proteins were measured in the RPPA platform. As shown in Fig. S1 (Additional file 3), we observed no significant differences.

We further investigated the oncoprint plots of the most significant biomarkers to determine whether they were co-expressed locally in a subset of the samples. We defined samples as “highly expressed” if the expression of a given biomarker in these samples was in the highest quartile of the expression range, and we defined samples as “poorly expressed” if the expression value was in the lowest quartile. The analysis was performed for mRNA expression and protein expression data, respectively. As shown in Fig. 2, we created oncoprints of the top ten most significant protein biomarkers (Fig. 2c) and mRNA biomarkers (Fig. 2d). These oncoprints illustrated near-random distribution in the samples, indicating that each biomarker defined its own set of highly and poorly expressed samples and had weak overlap with the expression patterns of other biomarkers. These results confirmed that the 52 genes we observed represented unique prognostic biomarkers at both the mRNA-expression and protein-expression level and that they were not duplicated or redundant.

### Screening for stage-specific KIRC biomarkers

Tumor stage is one of the most important clinicopathologic factors associated with patient outcome. To determine whether the biomarkers we had observed provided prognosis power beyond stage information, we performed stratified survival analyses for each stage of KIRC. We downloaded sample stage annotations from TCGA and grouped the KIRC samples into four stages: stage I (*n* = 210 samples), stage II (*n* = 41), stage III (*n* = 107), and stage IV (*n* = 78). In each of these stages, we applied the same analysis strategy in searching for biomarkers using both mRNA and protein expression. We referred to the biomarkers that were significant in particular stages as stage-specific biomarkers.

### Stage-specific biomarkers using protein expression

At FDR < 0.05, we observed five protein biomarkers in stage I, four in stage III, and 18 in stage IV. We found no significant protein biomarkers in stage II, although there were two antibodies (YAP_pS127 and STAT5-alpha) with marginal significance (nominal *p =* 7.5×10^−4^ and *p*_adjust_ = 0.083 for YAP_pS127; nominal *p* = 8.9×10^−4^ and *p*_adjust_ = 0.083 for STAT5-alpha). A detailed list of the results is presented in (Additional file 4: Table S3). To qualify more biomarkers for analysis, we then employed a less-stringent criterion, selecting biomarkers if their nominal *p* values obtained by the log-rank test were less than 0.001. We noticed that this threshold was arbitrary, so we performed the subsequent analyses with caution. With nominal *p* < 0.001, we identified two protein biomarkers in the stage I samples (HSP70 and Rad51), two in stage II (YAP_ps127 and STAT5-alpha), four in stage III (GAB2, MIG-6, MAPK_pT202_Y204, and PEA-15), and seven in stage IV (TIGAR, FASN, AR, S6, ACC1, Cyclin_B1, and GATA3) (Table 2A). No significant biomarker was shared in more than one stage, although some were significant in one stage and barely missed being significant in a second stage. For example, PEA-15 (encoding gene: *PEA15*) was prognostic in stage III (nominal *p =* 5.0×10^−4^) but marginally missed our criterion in stage IV (nominal *p =* 0.002). Another biomarker, AR (encoding gene: *AR*), was prognostic in stage IV (nominal *p =* 3.0×10^−5^) and had a nominal *p =* 0.012 in stage III.

**Table 2:** Statistics of survival time prediction of KIRC stage-specific protein biomarkers

Protein	Gene	All stages			Stage I			Stage II			Stage III			Stage IV		
		p*	FDR	beta	p*	FDR	beta	p*	FDR	beta	p*	FDR	beta	p*	FDR	beta
HSP70	HSPA1A	0.20	0.27	0.11	*4.3 × 10* ^*−4*^	0.05	0.59	0.72	0.97	0.11	0.62	0.79	−0.08	0.89	0.95	−0.02
Rad51	RAD51	3.0 × 10^−7^	4.0 × 10^−6^	1.25	*8.9 × 10* ^*−4*^	0.05	1.93	0.35	0.93	1.0	0.06	0.41	0.70	0.03	0.16	1.07
YAP_pS127	YAP1	3.8 × 10^−5^	2.8 × 10^−4^	0.67	0.24	0.61	0.48	*7.5 × 10* ^*−4*^	0.08	2.18	0.07	0.41	0.42	0.21	0.42	0.40
STAT5-alpha	STAT5A	0.18	0.26	−0.20	0.90	0.96	0.04	*8.9 × 10* ^*−4*^	0.08	−2.76	0.08	0.41	−0.40	0.19	0.39	−0.31
GAB2	GAB2	2.3 × 10^−10^	4.1 × 10^−8^	−0.67	6.9 × 10^−3^	0.12	−0.55	0.49	0.93	−0.31	*4.5 × 10* ^*−5*^	5.9 × 10^−3^	−0.78	0.06	0.22	−0.40
MIG-6	ERRFI1	4.2 × 10^−10^	4.1 × 10^−8^	−1.12	0.04	0.23	−0.67	0.59	0.93	−0.43	*6.3 × 10* ^*−5*^	5.9 × 10^−3^	−1.46	0.09	0.29	−0.55
MAPK_ pT202_Y204*	MAPK1, MAPK3	2.3 × 10^−8^	5.8 × 10^−7^	−0.53	0.57	0.80	−0.11	0.60	0.93	−0.20	*3.0 × 10* ^*−4*^	0.019	−0.65	0.05	0.20	−0.33
PEA-15	PEA15	2.6 × 10^−8^	5.8 × 10^−7^	1.41	0.45	0.74	−0.41	0.03	0.52	1.87	*5.0 × 10* ^*−4*^	0.023	1.59	2.2 × 10^−3^	0.037	1.38
TIGAR	C12ORF5	1.2 × 10^−3^	4.5 × 10^−3^	0.90	0.07	0.33	1.43	0.63	0.93	0.89	0.89	0.94	−0.06	*1.3 × 10* ^*−5*^	1.9 × 10^−3^	2.74
FASN	FASN	2.0 × 10^−6^	2.1 × 10^−5^	0.91	0.87	0.96	0.07	0.30	0.93	0.80	0.34	0.58	0.34	*2.3 × 10* ^*−5*^	1.9 × 10^−3^	1.29
AR	AR	9.5 × 10^−10^	5.9 × 10^−8^	−1.22	1.3 × 10^−3^	0.05	−1.34	0.92	0.99	−0.07	0.01	0.21	−0.92	*3 × 10* ^*−5*^	1.9 × 10^−3^	−1.19
S6	RPS6	3.7 × 10^−4^	1.7 × 10^−3^	0.70	0.75	0.89	0.12	0.86	0.97	0.14	0.66	0.81	−0.15	*8.8 × 10* ^*−5*^	4.1 × 10^−3^	1.38
ACC1	ACACA	9.1 × 10^−9^	2.8 × 10^−7^	0.88	5.1 × 10^−3^	0.10	1	0.86	0.97	−0.13	0.19	0.46	0.35	*3.6 × 10* ^*−4*^	0.01	0.86
Cyclin_B1	CCNB1	5.9 × 10^−7^	7.3 × 10^−6^	0.60	0.40	0.71	0.32	0.37	0.93	0.37	0.70	0.82	0.10	*7.1 × 10* ^*−4*^	0.02	0.63
GATA3	GATA3	0.02	0.04	0.54	0.51	0.79	0.32	0.49	0.93	0.59	0.17	0.45	0.52	*7.4 × 10* ^*−4*^	0.02	1.07

*p-values were obtained using the log-rank test

*p*-valu﻿e in italic indicates the record was significant (nominal *p* < 0.001)

**Fig. 3:**
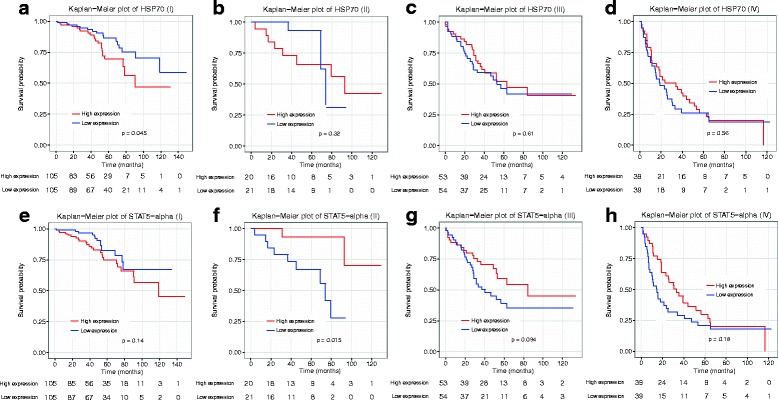
Kaplan–Meier plots of HSP70 and STAT5-ALPHA. HSPA1A (encoding gene: *HSPA1A*) is specifically prognostic in KIRC tumor stage I, while STAT5-alpha (encoding gene: *STAT5A*) is prognostic in KIRC stage II. The Kaplan-Meier plots of HSPA1A in samples from stages I-IV (**a, b, c,** and **d**, respectively) were compared. A similar comparison was made for STAT5-alpha (**e, f, g**, and **h**, respectively). The numbers at the bottom of plots show the number of samples at risk at each time point

We used two example genes to illustrate the stage-specific protein biomarkers (Fig. 3). Hsp70 is a heat-shock-response protein encoded by *HSPA1A*. Deregulated expression of Hsp70 at the protein level has been reported in KIRC [25]. In our work, we found that the protein expression of Hsp70 significantly predicts survival status for stage I samples of KIRC (Fig. 3a), but not for any of the other stages (Fig. 3b, c, and d), nor for all samples (*p =* 0.709). A second biomarker, STAT5-alpha (encoding gene *STAT5A*), was prognostic for survival in stage II (Fig. 3f), but not for other stages (Fig. 3e–or h).

**Fig. 4:**
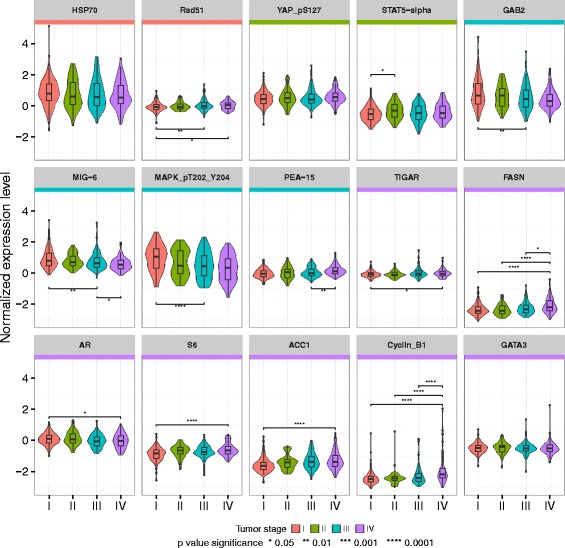
Protein expression of the 15 stage-specific KIRC biomarkers by stage. The Wilcoxon rank sum test was used to test the difference in expression level at each stage. The color bands on top of each plot show the stage in which the biomarker was prognostic. Statistical significance levels are marked by * (<0.05), ** (<0.01), *** (<0.001), and **** (<0.0001)

Next, we examined the distributions of protein expression, by stage, for each of the 15 stage-specific biomarkers we had identified. Our goal was to identify expression patterns that might explain the stage-specific prognostic capability of these biomarkers. Other than HSP70, YAP_pS127, and GATA3, all of the biomarkers displayed stage-specific expression changes (Fig. 4). For example, the protein expression level of Rad51 was significantly decreased in stage I compared to stage III and stage IV, and FASN’s protein expression level was significantly increased in stage IV compared to the other three stages. A similar pattern was observed in the other biomarkers; their protein expression differed in at least two different stages.

Using the PMC sample group, we screened for mRNA biomarkers and discovered 15 that were stage-specific and prognostic (nominal *p* < 0.001, log-rank test). Compared with the stage-specific protein biomarkers, mRNA biomarkers were surprisingly deficient in the early stages of KIRC (stages I, II, and III) but were abundant in stage IV (Additional file 5). As shown in Table 3, no significant mRNA biomarker was found in stage I or stage II, and only one gene, *KDR*, was significant in stage III (nominal *p =* 7.3×10^−4^). In contrast, in stage IV we found a total of 15 mRNA biomarkers, including *KDR*.

**Table 3:** Statistics of survival time prediction of KIRC stage-specific mRNA biomarkers

Gene	Protein	All stages			Stage I			Stage II			Stage III			Stage IV		
		*p* ^*^	FDR	beta	*p* ^*^	FDR	beta	*p* ^*^	FDR	beta	*p* ^*^	FDR	beta	*p* ^*^	FDR	beta
*KDR*	VEGFR2	6.4 × 10^−12^	2.4 × 10^−10^	−0.39	0.09	0.42	−0.24	0.72	0.87	0.10	*7.3 × 10* ^*−4*^	0.08	−0.35	4.3 × 10^−4^	7.5 × 10^−3^	−0.33
*FASN*	FASN	9.6 × 10^−12^	2.9 × 10^−10^	0.82	0.07	0.40	0.57	0.08	0.59	0.88	7.0 × 10^−3^	0.08	0.53	*9.3 × 10* ^*−6*^	7.5 × 10^−4^	0.90
*FOXM1*	FoxM1	1.1 × 10^−12^	7.8 × 10^−11^	0.49	0.10	0.42	0.32	0.15	0.71	0.40	0.13	0.35	0.18	*1.0 × 10* ^*−5*^	7.5 × 10^−4^	0.51
*CCNE1*	Cyclin_E1	9.7 × 10^−14^	1.4 × 10^−11^	0.58	0.02	0.33	0.46	0.03	0.52	0.76	0.09	0.30	0.23	*3.4 × 10* ^*−5*^	1.7 × 10^−3^	0.54
*ERBB3*	HER3_pY128; HER3	1.4 × 10^−7^	1.1 × 10^−6^	−0.28	0.01	0.30	−0.34	0.24	0.78	−0.21	0.10	0.30	−0.18	*2.0 × 10* ^*−4*^	6.2 × 10^−3^	−0.25
*CCNE2*	Cyclin_E2	3.2 × 10^−5^	1.4 × 10^−4^	0.47	0.88	0.98	0.04	0.09	0.59	0.69	0.85	0.92	0.04	*2.5 × 10* ^*−4*^	6.2 × 10^−3^	0.70
*BCL2*	Bcl-2	4.9 × 10^−8^	4.5 × 10^−7^	−0.42	0.05	0.39	−0.30	0.62	0.87	0.16	0.04	0.18	−0.32	*2.5 × 10* ^*−4*^	6.2 × 10^−3^	−0.48
*ACACA*	ACC_pS79; ACC1	2.8 × 10–5	1.3 × 10^−4^	0.61	0.05	0.39	0.61	0.65	0.87	0.24	0.04	0.19	0.53	*4.3 × 10* ^*−4*^	7.5 × 10^−3^	0.82
*DVL3*	Dvl3	1.4 × 10^−11^	3.3 × 10^−10^	1.22	0.01	0.30	1.14	0.01	0.52	1.40	7.5 × 10^−3^	0.08	0.84	*4.6 × 10* ^*−4*^	7.5 × 10^−3^	1.09
*COL6A1*	Collagen_VI	1.5 × 10^−9^	2.5 × 10^−8^	0.50	0.21	0.71	0.28	0.18	0.71	0.30	5.4 × 10^−3^	0.08	0.45	*5.6 × 10* ^*−4*^	8.0 × 10^−3^	0.41
*CCNB1*	Cyclin_B1	6.9 × 10^−8^	6.0 × 10^−7^	0.48	0.49	0.86	0.16	0.35	0.80	0.34	0.81	0.90	0.04	*6.0 × 10* ^*−4*^	8.0 × 10^−3^	0.47
*RAD51*	Rad51	5.6 × 10^−5^	2.2 × 10^−4^	0.38	0.96	1.00	−0.01	0.14	0.71	0.42	0.42	0.66	−0.14	*7.9 × 10* ^*−4*^	9.4 × 10^−3^	0.55
*RB1*	Rb_pS807_S811	2.9 × 10^−6^	1.8 × 10^−5^	−0.68	0.66	0.89	−0.19	0.05	0.52	−1.59	3.4 × 10^−3^	0.08	−0.60	*8.7 × 10* ^*−4*^	9.4 × 10^−3^	−0.91
*VHL*	VHL	.07	0.11	0.22	0.34	0.84	0.26	0.18	0.71	0.61	0.53	0.74	−0.12	*9.1 × 10* ^*−4*^	9.4 × 10^−3^	0.69
*MYH11*	MYH11	8.5 × 10^−4^	2.5 × 10^−3^	−0.16	0.38	0.85	−0.10	0.97	1.00	−7.4 × 10^−3^	0.16	0.38	−0.13	*9.8 × 10* ^*−4*^	9.4 × 10^−3^	−0.22

*p-values were obtained using the log-rank test

**Fig. 5:**
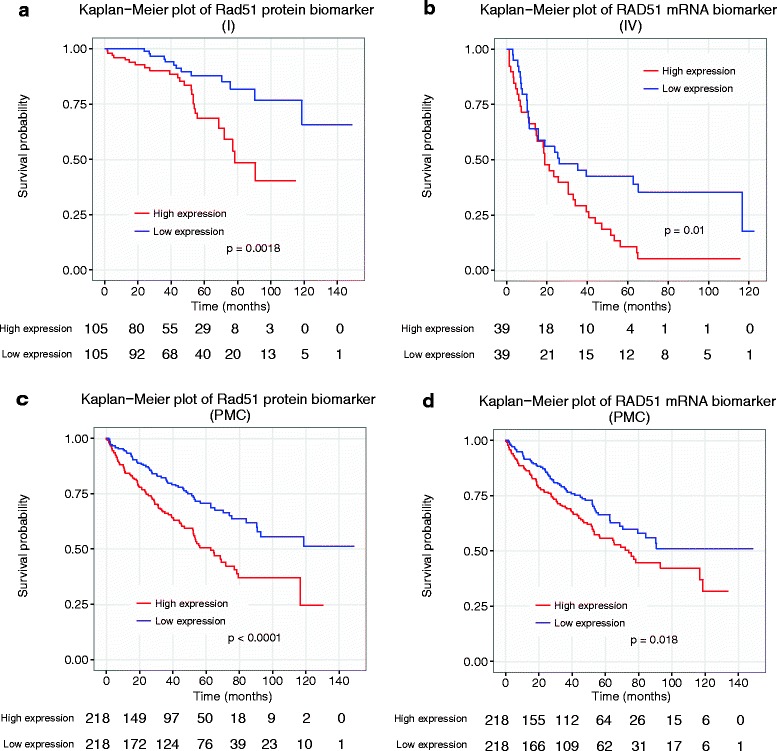
Kaplan–Meier plots of two stage-specific biomarkers. *RAD51* (corresponding antibody: Rad51) is a prognostic biomarker according to stage I protein data **a** and stage IV mRNA data (**b**). Kaplan-Meier plots of *RAD51* and the corresponding antibody were also examined using PMC samples (**c, d**). Numbers at the bottom of each plot show the number of samples at risk at each time point

Only four genes were associated with both stage-specific protein biomarkers and mRNA biomarkers: *RAD51* (antibody, Rad51; stage I protein biomarker; stage IV mRNA biomarker, Fig. 5), *FASN* (antibody FASN; protein and mRNA biomarkers for stage IV), *ACACA* (antibody ACC1; protein and mRNA biomarkers for stage IV), and *CCNB1* (antibody Cyclin_B1; protein and mRNA biomarkers for stage IV).

### Stage-specific biomarkers compared to general biomarkers

Next, we compared the 15 stage-specific protein biomarkers and the 15 stage-specific mRNA biomarkers with the 52 genes shared between the protein and mRNA biomarkers. As a result, we found seven stage-specific protein biomarkers among the 52 general biomarkers: ACC1, Cyclin_B1, FASN, GATA3, GAB2, MAPK_pT202_Y204, and Rad51. In addition, we found nine stage-specific mRNA biomarkers among the 52 general biomarkers: *ACACA*, *BCL2*, *CCNB1*, *CCNE1*, *DVL3*, *ERBB3*, *FASN*, *RAD51*, and *RB1*.

Alterations in DNA are linked to protein or mRNA changes. Therefore, to determine whether DNA alterations can predict patient outcomes, we searched for somatic mutations occurring in genes. In KIRC, several well-studied genes, such as *VHL*, *BAP1*, *PBRM1*, and *STAG2*, are associated with frequent somatic, nonsynonymous mutations [15]. However, in our analysis, none of these mutations demonstrated significant prognostic power. Previous studies have reported similar results, although they reported that *VHL* displayed modest prognostic power [26]. In our work, we found *VHL* as a stage-specific mRNA biomarker in stage IV (*p =* 0.001, Table 3), where samples with high mRNA expression of *VHL* were associated with worse outcome. However, when we examined all samples regardless of stage, we determined that *VHL* could not predict patient outcomes (*p =* 0.1). Among the four genes shared by stage-specific protein biomarkers and mRNA biomarkers, only the biomarker ACC1 (gene: *ACACA*) had sufficient mutation data for survival analysis; that is, no other biomarker had at least five samples with mutations. However, our analysis indicated that ACC1 has no prognosis power, even though both its mRNA and protein expressions were significant prognostic biomarkers for stage IV KIRC (Additional file 6).

## Discussion

In this study, we systematically screened potential protein biomarkers using TCGA data for ten different cancer types. Strikingly, among all the cancer types we examined, KIRC was the only one that showed many survival biomarkers in both protein-expression and mRNA-expression data. Although to date, predicting the survival of KIRC patients using somatic mutation biomarkers has been only modestly successful [26], our study indicated that using protein biomarkers to predict KIRC survival is promising.

YAP_ps127 is a stage II-specific biomarker identified in our analysis. The corresponding gene, *YAP1*, is a tumor-suppressor gene that plays a role in prostate cancer [27–30]. STAT5-alpha (gene: *STAT5A*) is another stage II-specific biomarker. Its corresponding protein participates in the signal transduction process by mediating cellular response to ERBB4 [31]. *STAT5A*’s involvement in many cancers, including prostate cancer [32], oral squamous cell carcinoma [33, 34], breast cancer [34], and colorectal cancer [35], has been reported. Gab2 (gene: *GAB2*) is an adaptor protein that is important for cancer-signaling transduction processes, including ERK signaling and PI3K-AKT signaling [36]. PEA-15 is a stage III-specific biomarker; its corresponding gene, *PEA15*, is involved in cell proliferation and apoptosis, and an ovarian cancer study showed that this gene is a promising target for cancer treatment [37]. The stage IV-specific biomarker FASN is a protein that is involved in cellular fatty acid metabolism and that is reportedly involved in different cancer types, including ovarian cancer and breast cancer [38].

The most significantly enriched KEGG pathway for the 52 general prognostic biomarkers was prostate cancer. The renal clear cell carcinoma pathway (hsa05211) was not observed on the enriched pathways list. We manually checked the individual biomarkers that were involved in the renal clear cell carcinoma pathway, but only seven of 148 genes investigated in this study were found in the renal clear cell carcinoma pathway. This may explain why the pathway was not included on the list of enriched pathways.

Although our study reported a number of candidate protein and mRNA biomarkers specific to KIRC, it had several limitations. The first limitation is the existence of covariates. Covariates data is important for constructing a reliable prognostic model [39]. In our stage-specific biomarker analysis, there were more samples from male patients than from female patients. In addition, the age of the female patients was greater than that of the male patients (Fig. 1c). Due to the limited sample size, there was insufficient data available for us to take these and other influential factors such as living habitation and race into consideration. Since our study was a large-scale exploration, we attempted to use limited samples to find potential but strong trends. However, in order to draw more robust conclusions, future studies should include more independent samples. With an increased sample size, the covariates mentioned above could be carefully considered.

The second limitation of this study is that the proteins we investigated were restricted to those assayed in an RPPA array. Although these proteins were carefully selected and usually play important roles in cancer pathologies, it is possible that many other proteins that were not included in the RPPA array are also prognostic for KIRC survival. Wang et al. ‘s [40] study, which involved screening for mRNA prognostic biomarkers, supports this possibility. In our study, we tested different molecular types for the prognostic power. The prognostic mRNAs we identified are involved in acute-phase responses, death-receptor signaling, and the inhibition of matrix metalloproteases, and the proteins we identified include representative proteins involved in apoptosis, the cell cycle, the repair of DNA damage, and PI3K/AKT/mTOR signaling. Based on the fact that information flows from transcriptome to proteome, additional prognostic proteins involved in the inhibition of matrix metalloproteases may exist. Accordingly, further large-scale screenings of potential protein biomarkers are necessary so that more significant prognostic protein biomarkers can be identified.

The third limitation of our study is that we were restricted in the types of cancer we could include. We observed that, among the cancer types we examined, there are many prognostic protein biomarkers only in KIRC. However, because of the rapid accumulation of multi-omics data for many other cancer types (i.e., the 50 cancer types or subtypes that will be analyzed in the International Cancer Genome Consortium project), we will soon be able to seek biomarkers in other cancer types/subtypes; thus, the statement that there are many prognostic protein biomarkers only in KICR may need to be revised. Finally, our study only included protein-expression and gene-expression data. It is possible that data on methylation and noncoding RNA (microRNA or long noncoding RNA) have now become available for KIRC. Gene regulation is a complex and dynamic process, and with the inclusion of additional data types, we will gain a deeper understanding of biomarker function and regulation in KIRC tumorigenesis. This knowledge, in turn, will lead to the development of better therapeutic strategies for specific subgroups of patients with KIRC. With the rapid accumulation of multi-omics data, a comprehensive investigation of the transcriptome and proteome changes in KIRC may serve as the first step to reveal the mechanisms underlying these biomarkers.

Mutation resolution is important in biomarker screening. For the mutation biomarker screening analysis in KIRC, we also screened the mutation biomarkers with mutation types taken into consideration. Because the number of samples available would decrease for a particular mutation type, we only considered 10 genes which were most frequently mutated in KIRC. Chi-square test was used to identify prognostic biomarkers. For each gene in the analysis, we required ≥5 samples to carry the mutations of each type. With nominal *p* value threshold at 0.01, frameshift deletions in *SETD2* was found to be significant (*p* = 4.88 × 10^−3^).

The results we presented in this work required further validation in independent datasets. Currently, TCGA is the only data source that provide both mRNA-expression data and protein-expression data for KIRC. With the rapid accumulation of multi-omics data for cancer, we expected that more data would be available and future studies would warrant the validity of our work.

## Conclusions

Our pan-cancer screening revealed a surprisingly large number of protein biomarkers that were prognostic for survival in patients with KIRC. This large number of biomarkers was similarly observed at the mRNA biomarker level, but not at the DNA mutation level. Additionally, this feature was observed in KIRC, but not in other common types of cancer. Furthermore, several stage-specific KIRC biomarker candidates were identified and discussed. In summary, our study suggests that protein-level biomarkers could potentially have clinical value in determining the prognosis for KIRC patients and in developing treatment strategies based on tumor stage.

## Methods

### Data collection

Preprocessed mutation data were retrieved from the TumorPortal [41]. Both normalized RPPA data and normalized mRNA-expression data were downloaded from the UCSC Cancer Browser [42]. For the RPPA protein expression data, we used the dataset that was released on August 28, 2014 (file name: TCGA-PANCAN11-RBN.csv). The data had been preprocessed using the replicates-based normalization method [43]. For the mRNA expression data, we used the dataset that was released on January 28, 2015 (file name: TCGA_PANCAN12_exp_HiSeqV2–2015–01-28.tar.gz). The clinical data were downloaded from the cBio Cancer Genomics Portal [44] using the R package *cgdsr*. For each cancer type, we obtained the overall survival (in months) of the patients from whom the corresponding samples were taken.

### Screening for general prognostic biomarkers in ten types of cancer

#### Analysis of mutation data

For each cancer type, we included two somatic mutation types: insertion/deletion (indel) and SNV. Specifically, for indel mutations, we included all the available indel mutations except for silent indel mutations, and for SNVs, we excluded all the synonymous or silent SNVs. For our mutation analysis, we required ≥60 samples for each cancer type, and all ten cancer types fulfilled this criterion. We used the chi-square test to identify prognostic biomarkers in the filtered data. For each gene in the analysis, we required ≥5 samples carrying the mutation(s). Statistical *p* values were adjusted by the Benjamini and Hochberg method [23]. The FDR threshold was set to 0.05 to identify significant prognostic genes for the mutation analysis.

#### Analysis of RPPA and mRNA expression data

For each of the ten cancer types, we first identified three sample groups (Fig. 1a). The PC group had both RPPA protein-expression and clinical data, the MC group had both mRNA-expression and clinical data, and the PMC group had protein-expression, mRNA-expression, and clinical data. For the protein biomarker screening, we included 187 proteins. We mapped protein IDs to gene symbols using the RPPA protein-annotation file provided in Li et al. [2]. One hundred and forty-eight corresponding genes were used in the mRNA biomarker screening. For each protein or gene, we applied the Cox proportional hazards regression model using the *coxph* function in the R package *survival*. Multiple testing corrections were performed using Benjamini and Hochberg’s method. Unless otherwise stated, we used an FDR threshold of 0.05 to define significant biomarkers.

### Screening for stage-specific prognostic biomarkers in KIRC

KIRC stage-specific prognostic biomarkers were screened using both protein-expression and mRNA-expression data. The samples in the PMC sample group were used to screen the stage-specific protein and mRNA biomarkers, and since both kinds of biomarkers were screened using the same sample set, the results were more comparable. Samples were categorized by their stage annotation, which was defined based on the American Joint Committee on Cancer sample stage information and available in the clinical data. For example, our main stage I included the American Joint Committee on Cancer’s stage I, stage IA, and stage IB. We initially had five main stages for KIRC. For the follow up analyses, however, we excluded any main stages with sample sizes ≤3. This process removed main stage IV, so we only analyzed the data for the remaining four main stages.

For each RPPA protein or corresponding gene, we performed survival analyses using *coxph* in the R package *survival*. The *p* values from the Cox proportional hazards regression log- rank test were corrected for multiple testing using Benjamini and Hochberg’s method [23]. The significance threshold was set to 0.05 for the FDR values, and with this threshold, we found that there were no significant biomarkers for stage II KIRC. A less-stringent criterion was then used in the analysis (nominal log-rank test *p* value <0.001). Significant, stage-specific prognostic markers were used for downstream analysis.

To perform the enrichment analysis of the KIRC protein and mRNA biomarkers, we converted the protein biomarkers into gene symbols and used genes as the basis for the enrichment analysis. We used a hypergeometric test to assess the enrichment level, as we had done in previous studies [45–47].

### Analysis of co-expression in general prognostic biomarkers

We used the PCC to assess the coregulation of the 52 general prognostic biomarkers. We obtained the gene symbols corresponding to the 148 RPPA proteins and extracted their protein expression profiles. When multiple protein biomarkers were mapped to one gene, one representative protein biomarker was randomly chosen. Pairwise gene correlation was calculated for the protein expression profile, and samples with missing values were excluded from this step of PCC computation. We defined the distance as one minus the absolute value of the PCC and performed hierarchical clustering of the candidate biomarkers. The PCC for mRNA expression was calculated the same way and is plotted in the lower triangle of the heatmap in Fig. 2b.

Oncoprint plots were used to confirm whether the most significant prognostic biomarkers clustered together in a subset of samples. We plotted oncoprints for both the protein-expression and the mRNA-expression data. For each protein or each gene, the 25% quartile and the 75% quartile of the gene-expression profile were calculated using data for the entire sample set. Samples with expression values >75% quantile were labeled as highly expressed, while samples with expression values <25% quantile were labeled as poorly expressed. Based on this procedure, we developed an in-house R script to generate oncoprint plots. The script is available upon request.

## Additional files

Additional file 1: Table S1. Statistical summary of KIRC protein and mRNA biomarkers prognostic for survival (XLSX 19 kb)

Additional file 2: Table S2. Gene set enrichment analysis results for protein biomarkers prognostic for KIRC survival (XLSX 16 kb)

Additional file 3: Figure S1. Comparison of protein-expression and mRNA-expression correlations between prognostic proteins biomarkers and non-prognostic proteins (PDF 15 kb)

Additional file 4: Table S3. Screening results for stage-specific RPPA protein biomarkers prognostic for KIRC (XLSX 61 kb)

Additional file 5: Table S4. Screening results for stage-specific mRNA biomarkers prognostic for KIRC (XLSX 49 kb)

Additional file 6: Figure S2. Kaplan–Meier (KM) plots of ACC1 protein data (Fig. S2A) and mutation data (Fig. S2B) (PDF 39 kb)

## References

[1] Siegel RL, Miller KD, Jemal A (2016). Cancer statistics, 2016. CA Cancer J Clin.

[2] Li J, Lu Y, Akbani R, Ju Z, Roebuck PL, Liu W, Yang JY, Broom BM, Verhaak RG, Kane DW (2013). TCPA: a resource for cancer functional proteomics data. Nat Methods.

[3] Korpanty GJ, Graham DM, Vincent MD, Leighl NB (2014). Biomarkers that currently affect clinical practice in lung cancer: EGFR, ALK, MET, ROS-1, and KRAS. Front Oncol.

[4] Renaud S, Romain B, Falcoz PE, Olland A, Santelmo N, Brigand C, Rohr S, Guenot D, Massard G (2015). KRAS and BRAF mutations are prognostic biomarkers in patients undergoing lung metastasectomy of colorectal cancer. Br J Cancer.

[5] Weinstein D, Leininger J, Hamby C, Safai B (2014). Diagnostic and prognostic biomarkers in melanoma. J Clin Aesthet Dermatol.

[6] Chen HY, Yu SL, Chen CH, Chang GC, Chen CY, Yuan A, Cheng CL, Wang CH, Terng HJ, Kao SF (2007). A five-gene signature and clinical outcome in non-small-cell lung cancer. N Engl J Med.

[7] Karimi S, Mohamadnia A, Nadji SA, Yadegarazari R, Khosravi A, Bahrami N, Saidijam M (2015). Expression of two basic mRNA biomarkers in peripheral blood of patients with non-small cell lung cancer detected by real-time rt-PCR, individually and simultaneously. Iran Biomed J.

[8] Kishikawa T, Otsuka M, Ohno M, Yoshikawa T, Takata A, Koike K (2015). Circulating RNAs as new biomarkers for detecting pancreatic cancer. World J Gastroenterol.

[9] Nilsson J, Skog J, Nordstrand A, Baranov V, Mincheva-Nilsson L, Breakefield XO, Widmark A (2009). Prostate cancer-derived urine exosomes: a novel approach to biomarkers for prostate cancer. Br J Cancer.

[10] Li Y, St John MA, Zhou X, Kim Y, Sinha U, Jordan RC, Eisele D, Abemayor E, Elashoff D, Park NH (2004). Salivary transcriptome diagnostics for oral cancer detection. Clin Cancer Res.

[11] Li Y, Elashoff D, Oh M, Sinha U, St John MA, Zhou X, Abemayor E, Wong DT (2006). Serum circulating human mRNA profiling and its utility for oral cancer detection. J Clin Oncol.

[12] Spurrier B, Ramalingam S, Nishizuka S (2008). Reverse-phase protein lysate microarrays for cell signaling analysis. Nat Protoc.

[13] Jemal A, Siegel R, Xu J, Ward E (2010). Cancer statistics, 2010. CA Cancer J Clin.

[14] Chow WH, Dong LM, Devesa SS (2010). Epidemiology and risk factors for kidney cancer. Nat Rev Urol.

[15] The Cancer Genome Atlas Research Network (2013). Comprehensive molecular characterization of clear cell renal cell carcinoma. Nature.

[16] Guo G, Gui Y, Gao S, Tang A, Hu X, Huang Y, Jia W, Li Z, He M, Sun L (2012). Frequent mutations of genes encoding ubiquitin-mediated proteolysis pathway components in clear cell renal cell carcinoma. Nat Genet.

[17] Sato Y, Yoshizato T, Shiraishi Y, Maekawa S, Okuno Y, Kamura T, Shimamura T, Sato-Otsubo A, Nagae G, Suzuki H (2013). Integrated molecular analysis of clear-cell renal cell carcinoma. Nat Genet.

[18] Gerlinger M, Horswell S, Larkin J, Rowan AJ, Salm MP, Varela I, Fisher R, McGranahan N, Matthews N, Santos CR (2014). Genomic architecture and evolution of clear cell renal cell carcinomas defined by multiregion sequencing. Nat Genet.

[19] Wang Y, Bray M, Guo X, Ding Z, Zhao Z (2016). Integrated genomics analyses revealed novel functional consequences of PBRM1 truncated mutations in clear-cell renal cell carcinoma. BMC Genomics.

[20] Varela I, Tarpey P, Raine K, Huang D, Ong CK, Stephens P, Davies H, Jones D, Lin ML, Teague J (2011). Exome sequencing identifies frequent mutation of the SWI/SNF complex gene PBRM1 in renal carcinoma. Nature.

[21] Jia P, Zhao Z. Impacts of somatic mutations on gene expression: an association perspective. Brief Bioinform. 2016; 10.1093/bib/bbw037 PMC5862283 27127206

[22] Cerami E, Gao J, Dogrusoz U, Gross BE, Sumer SO, Aksoy BA, Jacobsen A, Byrne CJ, Heuer ML, Larsson E (2012). The cBio cancer genomics portal: an open platform for exploring multidimensional cancer genomics data. Cancer Discov.

[23] Benjamini Y, Hochberg Y. Controlling the false discovery rate: a practical and powerful approach to multiple testing. J R Stat Soc Ser B Methodol. 1995:289–300.

[24] Wang K, Li M, Hakonarson H (2010). ANNOVAR: functional annotation of genetic variants from high-throughput sequencing data. Nucleic Acids Res.

[25] Shi T, Dong F, Liou LS, Duan ZH, Novick AC, DiDonato JA (2004). Differential protein profiling in renal-cell carcinoma. Mol Carcinog.

[26] Vasselli JR, Shih JH, Iyengar SR, Maranchie J, Riss J, Worrell R, Torres-Cabala C, Tabios R, Mariotti A, Stearman R (2003). Predicting survival in patients with metastatic kidney cancer by gene-expression profiling in the primary tumor. Proc Natl Acad Sci U S A.

[27] Levy D, Adamovich Y, Reuven N, Shaul Y (2008). Yap1 phosphorylation by c-Abl is a critical step in selective activation of proapoptotic genes in response to DNA damage. Mol Cell.

[28] Zhao B, Wei X, Li W, Udan RS, Yang Q, Kim J, Xie J, Ikenoue T, Yu J, Li L (2007). Inactivation of YAP oncoprotein by the hippo pathway is involved in cell contact inhibition and tissue growth control. Genes Dev.

[29] Zhao B, Ye X, Yu J, Li L, Li W, Li S, Yu J, Lin JD, Wang CY, Chinnaiyan AM (2008). TEAD mediates YAP-dependent gene induction and growth control. Genes Dev.

[30] Tomlinson V, Gudmundsdottir K, Luong P, Leung KY, Knebel A, Basu S (2010). JNK phosphorylates yes-associated protein (YAP) to regulate apoptosis. Cell Death Dis.

[31] Williams CC, Allison JG, Vidal GA, Burow ME, Beckman BS, Marrero L, Jones FE (2004). The ERBB4/HER4 receptor tyrosine kinase regulates gene expression by functioning as a STAT5A nuclear chaperone. J Cell Biol.

[32] Liao Z, Lutz J, Nevalainen MT (2010). Transcription factor Stat5a/b as a therapeutic target protein for prostate cancer. Int J Biochem Cell Biol.

[33] Kar P, Supakar PC (2006). Expression of Stat5A in tobacco chewing-mediated oral squamous cell carcinoma. Cancer Lett.

[34] Peck AR, Witkiewicz AK, Liu C, Klimowicz AC, Stringer GA, Pequignot E, Freydin B, Yang N, Ertel A, Tran TH (2012). Low levels of Stat5a protein in breast cancer are associated with tumor progression and unfavorable clinical outcomes. Breast Cancer Res.

[35] Hong X, Chen G, Wang M, Lou C, Mao Y, Li Z, Zhang Y (2012). STAT5a-targeting miRNA enhances chemosensitivity to cisplatin and 5-fluorouracil in human colorectal cancer cells. Mol Med Rep.

[36] Adams SJ, Aydin IT, Celebi JT (2012). GAB2--a scaffolding protein in cancer. Mol Cancer Res.

[37] Xie X, Bartholomeusz C, Ahmed AA, Kazansky A, Diao L, Baggerly KA, Hortobagyi GN, Ueno NT (2013). Bisphosphorylated PEA-15 sensitizes ovarian cancer cells to paclitaxel by impairing the microtubule-destabilizing effect of SCLIP. Mol Cancer Ther.

[38] Flavin R, Peluso S, Nguyen PL, Loda M (2010). Fatty acid synthase as a potential therapeutic target in cancer. Future Oncol.

[39] Burton A, Altman DG (2004). Missing covariate data within cancer prognostic studies: a review of current reporting and proposed guidelines. Br J Cancer.

[40] Yuan Y, Van Allen EM, Omberg L, Wagle N, Amin-Mansour A, Sokolov A, Byers LA, Xu Y, Hess KR, Diao L (2014). Assessing the clinical utility of cancer genomic and proteomic data across tumor types. Nat Biotechnol.

[41] Lawrence MS, Stojanov P, Mermel CH, Robinson JT, Garraway LA, Golub TR, Meyerson M, Gabriel SB, Lander ES, Getz G (2014). Discovery and saturation analysis of cancer genes across 21 tumour types. Nature.

[42] Cline MS, Craft B, Swatloski T, Goldman M, Ma S, Haussler D, Zhu J (2013). Exploring TCGA pan-cancer data at the UCSC cancer genomics browser. Sci Rep.

[43] Akbani R, Ng PK, Werner HM, Shahmoradgoli M, Zhang F, Ju Z, Liu W, Yang JY, Yoshihara K, Li J (2014). A pan-cancer proteomic perspective on the cancer genome atlas. Nat Commun.

[44] Gao J, Aksoy BA, Dogrusoz U, Dresdner G, Gross B, Sumer SO, Sun Y, Jacobsen A, Sinha R, Larsson E (2013). Integrative analysis of complex cancer genomics and clinical profiles using the cBioPortal. Sci Signal.

[45] Jia P, Zhao Z (2014). VarWalker: personalized mutation network analysis of putative cancer genes from next-generation sequencing data. PLoS Comput Biol.

[46] Guo AY, Webb BT, Miles MF, Zimmerman MP, Kendler KS, Zhao Z (2009). ERGR: an ethanol-related gene resource. Nucleic Acids Res.

[47] Jia P, Pao W, Zhao Z (2014). Patterns and processes of somatic mutations in nine major cancers. BMC Med Genet.

