# Supplementary figures and images for: Unique protein expression signatures of survival time in kidney renal clear cell carcinoma through a pan-cancer screening
Source: BMC Genomics. 2017 Oct 3;18(Suppl 6):678. doi: 10.1186/s12864-017-4026-6 (PMC5629613; doi:10.1186/s12864-017-4026-6)

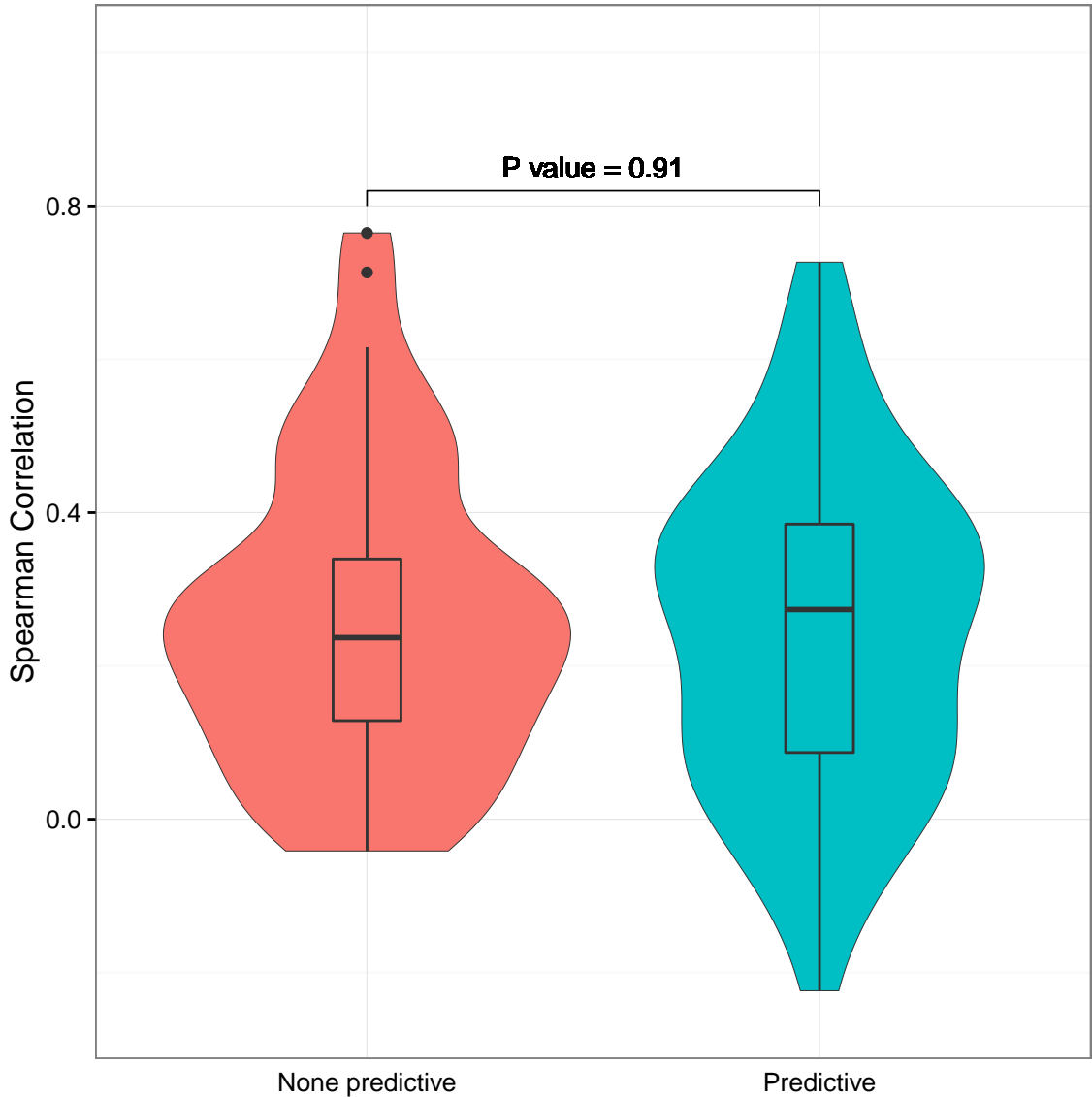

Supplement: Supplementary file 3 — Comparison of protein-expression and mRNA-expression correlations between prognostic proteins biomarkers and non-prognostic proteins (PDF 15 kb) [file 12864_2017_4026_MOESM3_ESM.pdf]
