# Supplementary material for: Unique protein expression signatures of survival time in kidney renal clear cell carcinoma through a pan-cancer screening
Source: BMC Genomics. 2017 Oct 3;18(Suppl 6):678. doi: 10.1186/s12864-017-4026-6 (PMC5629613; doi:10.1186/s12864-017-4026-6)
Supplement: Supplementary file 6 — Kaplan–Meier (KM) plots of ACC1 protein data (Fig. S2A) and mutation data (Fig. S2B) (PDF 39 kb) [file 12864_2017_4026_MOESM6_ESM.pdf]

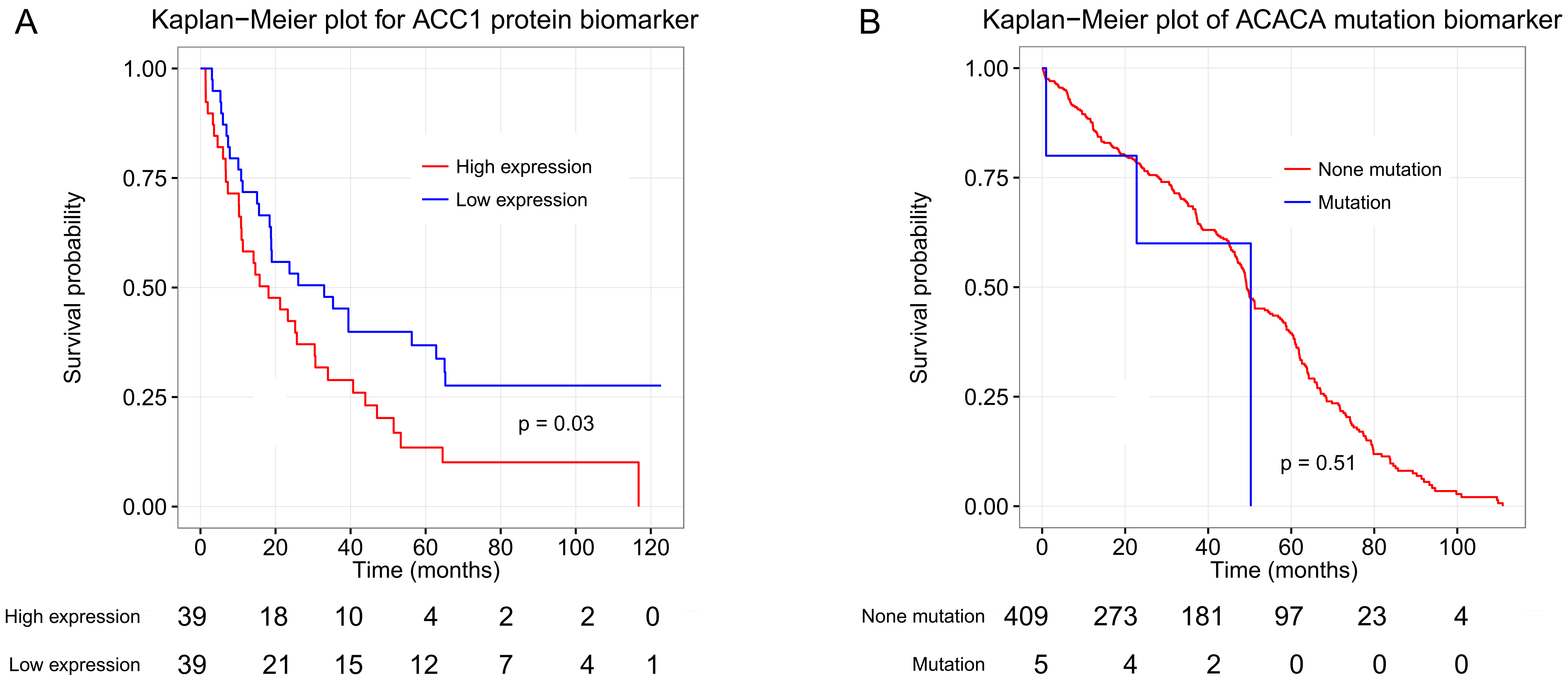
A
Kaplan−Meier plot for ACC1 protein biomarker
1.00
0.75
0.50
0.25
0.00
Survival probability
High expression
Low expression
p = 0.03
0
20
40
60
80
100
120
Time (months)
High expression 39 18 10 4 2 2 0
Low expression 39 21 15 12 7 4 1
B
Kaplan−Meier plot of ACACA mutation biomarker
1.00
0.75
0.50
0.25
0.00
Survival probability
None mutation
Mutation
p = 0.51
0
20
40
60
80
100
Time (months)
None mutation 409 273 181 97 23 4
Mutation 5 4 2 0 0 0
